# Intermittent hypoxia improves cognition and reduces anxiety‐related behavior in APP/PS1 mice

**DOI:** 10.1002/brb3.1513

**Published:** 2019-12-26

**Authors:** Sheng‐Xi Meng, Bing Wang, Wen‐Tao Li

**Affiliations:** ^1^ Shanghai Jiao Tong University Affiliated Sixth People’s Hospital Shanghai China; ^2^ Department of Vasculocardiology Municipal Hospital of Traditional Chinese Medicine Affiliated to Shanghai University of Traditional Chinese Medicine Shanghai China

**Keywords:** Alzheimer's disease, anxiety, APP/PS1 mice, intermittent hypoxia

## Abstract

**Introduction:**

Although hypoxia can exacerbate symptoms of various neurological disorders, accumulating evidence has indicated that intermittent hypoxia (IH) may exert protective effects against brain diseases. In the present study, we aimed to determine whether exposure to IH exerts beneficial effects in a transgenic murine model of Alzheimer's disease (AD). Because comorbid anxiety is prevalent among patients with AD, we explored the effects of IH on anxiety‐like behaviors and associated factors in APP/PS1 mice.

**Methods:**

APP/PS1 mice were subjected to IH for two weeks. We assessed cognitive performance and anxiety‐related behavior using standard behavioral assessments. Amyloid beta (Aβ) levels in the hippocampus were assessed using immunofluorescence and enzyme‐linked immunosorbent assays (ELISA). We also assessed cell morphology and brain‐derived neurotrophic factor (BDNF) expression in the hippocampus.

**Results:**

Exposure to IH significantly increased cognitive performance and decreased anxiety‐related behaviors in APP/PS1 mice. Immunofluorescence and ELISA results revealed that IH pretreatment significantly lowered Aβ levels in the cortex and hippocampus. Morphological studies validated the neuroprotective effect of IH exposure on hippocampal neurogenesis. Molecular studies revealed IH‐enhanced BDNF expression and inhibition of apoptosis‐related protein expression in the hippocampus of APP/PS1 mice.

**Conclusions:**

Our study demonstrates that IH improves cognition and reduces anxiety in a murine model of AD. Thus, further studies are required to determine whether IH can be used as a preventive/adjuvant therapy in patients with AD.

## Graphical abstract

It is the first to investigate the effects of IH exposure on spatial memory, cognition, and anxiety‐related behavior in the APP/PS1 mouse model of AD. IH improves cognition and reduces anxiety in a murine model of AD.

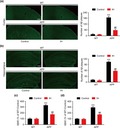

## 1 INTRODUCTION

Alzheimer's disease (AD) is characterized by the extracellular deposition of amyloid beta (Aβ) and the presence of intracellular neurofibrillary tangles, which act to induce neural apoptosis (Sonkar et al., 2016). AD is the leading cause of dementia, and its main clinical manifestations include gradual decreases in memory, cognitive dysfunction, and behavioral abnormalities (Rocca et al., 2011). Disorders affecting emotional regulation such as anxiety are common among patients with AD (Cassimjee, 2007). Symptoms of cognitive impairment and anxiety decrease the quality of life among patients with AD and increase the social and clinical burden of this disease.

Previous research has demonstrated a relationship between Aβ pathology and cognitive function decline in patients with AD (Insel et al., 2016). Inhibition of neurogenesis is closely associated with hippocampus‐mediated cognitive and memory dysfunction in the pathogenesis of AD, and both patients with AD and pathological models exhibit reduced numbers of neonatal neurons (Gouras & Fillit, 2006). These symptoms are thought to be closely related to the weakened ability of neural stem cells (NSCs) to regenerate and differentiate under the pathological conditions of AD. Aβ pathology also contributes to neuronal vulnerability and neurotoxicity (Li et al., 2015). Promoting neuronal regeneration is, therefore, an effective strategy for improving cognitive symptoms in patients with AD (Jin, Xie, Mao, & Greenberg, 2006).

Adult neurogenesis requires neurotrophic stimulation. Brain‐derived neurotrophic factor (BDNF) is widely expressed in the adult brain and plays a key role in neurogenesis, which has a profound impact on the development of AD (Avila, 2011; Yasutake, Kuroda, Yanagawa, Okamura, & Yoneda, 2006). Postmortem brain samples from patients with AD exhibit reduced levels of BDNF (Du et al., 2018). Moreover, increased signaling from neurotrophic factors such as BDNF exerts a neuroprotective effect in the aging brain, suggesting that neurogenesis is impaired in patients with AD (Liu, Xue, Shi, Qi, & Gong, 2015).

Previous studies have indicated that neuronal loss in postmortem brain samples from patients with AD occurs due to necroptosis (Padurariu, Ciobica, Mavroudis, Fotiou, & Baloyannis, 2012). Reducing necroptosis, in turn, decreases cell loss and helps to ameliorate cognitive dysfunction (Li et al., 2014). Studies of aberrant apoptotic protein expression have revealed that AD brains exhibit changes in cysteine protease expression (Monnerie & Le Roux, 2008). In addition to caspases, the proapoptotic protein Bax and the anti–apoptotic protein Bcl‐2 maintain a balanced antagonistic response to apoptotic stimuli (Nasiraei‐Moghadam, Kazeminezhad, Dargahi, & Ahmadiani, 2010).

According to a report by the Pharmaceutical Research and Manufacturers of America (PhRMA), 146 AD drugs failed between 1998 and 2017 in clinical practice worldwide, and only four have been marketed successfully. These drugs only provide symptomatic treatment and cannot alter the progression of the disease. The limitations of pharmacotherapy studies have encouraged the exploration of alternative therapies.

Chronic intermittent hypoxia (IH) mimics experimental models of sleep apnea, which has been suggested to exhibit a close relationship with neurodegenerative disorders including AD (Liu, Qiu, Yang, Ni, & Le, 2016; Snyder, Shell, Cunningham, & Cunningham, 2017). However, in brief, repeated adaption to moderate hypoxia has been shown to play a protective role in the brain (Lin, Chen, & Ho, 2002). For example, a previous study reported that IH preadaptation attenuates symptoms of memory impairment in AD rats (Manukhina et al., 2010). Moreover, IH exerts antidepressant‐like effects via enhanced expression of BDNF and neurogenesis in the rat hippocampus (Zhu et al., 2010). These observations indicate that IH may also exert a protective effect in AD by promoting hippocampal neurogenesis and BDNF expression. In the present study, we investigated the mechanisms underlying the effects of IH exposure in a mouse model of AD.

## 2 MATERIALS AND METHODS

### 2.1 Animals

Animal studies were approved by the Shanghai Jiao Tong University Institutional Animal Care and Use Committee (IACUC), and all experiments were conducted in accordance with the National Institute of Health guidelines for animal care. C57B6 male APP/PS1 mice (age: 9 months) were obtained from the Shanghai Laboratory Animal Management Centre. All transgenic animals were genotyped by performing a tail DNA polymerase chain reaction analysis prior to experiments. Mice were housed (no more than five animals per cage) under a 12‐hr light/dark cycle and provided ad libitum access to food and water until 2 weeks prior to behavioral testing, at which time they were housed individually. Behavioral tests were performed during the light phase.

### 2.2 Exposure to IH

For IH, a hypoxic tank consisting of a plexiglass chamber was purchased from Changjin Technology Co., Ltd. (Changsha, China). An oxygen concentration meter was attached to the side of the container. The mice were placed in the hypoxic pressure chamber for 4 hr per day for 15 consecutive days. During IH exposure, the oxygen pressure decreased to a height of 5 km at a speed of 30 m/s. After 4 hr of exposure, the pressure dropped to ground pressure at a speed of 30 m/s. During IH, experimental animals were deprived of any solid or liquid intake. The normoxic control mice were placed in the same plexiglass chamber with room air for the corresponding amount of time.

### 2.3 Morris water maze (MWM) test

Following IH exposure, the MWM test was performed as previously described (Bromley‐Brits, Deng, & Song, 2011). Briefly, the water temperature was adjusted to 22–24°C. The acquisition test was performed four times per day for five consecutive days. The time required to find the hidden platform was defined as the escape latency. The platform position was consistent for each mouse between trials. Retention tests were performed 1 day after the final acquisition session. The platform was removed, and mice were allowed to explore freely for 60 s. The time required and the number of crossings over the platform area were recorded. The entire experiment was recorded using a digital camera trace monitoring system (Noldus Information Technology, Wageningen, The Netherlands).

### 2.4 Spontaneous alternation (Y‐maze) test

The Y‐maze test was performed as previously described (Ma, Chen, He, Zeng, & Wang, 2007). Mice were placed into the center of the maze and allowed to explore freely across the three arms of the maze during an 8‐min session. The sequence of arm entries and the total number of arm entries were recorded. Spontaneous alternation performance (%) was calculated as follows: (entries into all three arms)/(total arm entries−2) × 100%.

### 2.5 Novel object recognition (NOR) test

In accordance with previously described methods (Carlini et al., 2008), mice were subjected to a 3‐day NOR test. Day 1 included the adaptation period, during which the mice were allowed to explore the box for 10 min. On day 2, two identical toys (4 cm × 4 cm) were placed into the box, and the time the mice spent exploring each object was recorded. Day 3 included the recognition period: Another object was placed into the box to replace one of the toys, and the total time the mice spent exploring each object was recorded. The discrimination score was calculated as follows:

Discrimination score = New Object Time/(New Object Time + Old Object Time)−Old Object Time/(New Object Time + Old Object Time).

### 2.6 Open‐field test (OFT)

Mice were placed in the center of the open‐field box, and behavioral changes were recorded for 30 min using a camera system. Experimental parameters included the distance traveled and the percentage of time spent in the central area.

### 2.7 Elevated plus‐maze (EPM) test

The EPM device was consistent with that described in previous reports (Itoh, Nabeshima, & Kameyama, 1991). A camera was installed directly above the cross maze, and the mice were placed in the center and allowed to move freely throughout the maze. Noldus software was used to track the location and record mouse behavior for 5 min.

### 2.8 Immunohistochemistry

Mice were deeply anesthetized and transcardially perfused with ice‐cold 0.9% saline, followed by 4% paraformaldehyde dissolved in 0.1 M phosphate‐buffered saline (PBS). The fixed, cryoprotected mouse brains were frozen and then cut into 35‐μm‐thick coronal sections. Free‐floating sections were washed three times in PBS, following which they were incubated for 1 hr in 5% normal goat serum in PBS containing 0.3% Triton X‐100. Sections were incubated overnight at 4°C with the following primary antibodies: 6E10 mouse monoclonal anti‐Aβ (1:1,000, Chemicon; MAB1560), rat monoclonal anti‐bromodeoxyuridine (BrdU; 1:100, AbD Serotec; OBT0030), goat anti‐doublecortin (DCX; 1:200, Santa Cruz Biotechnology; SC8066), mouse anti‐glial fibrillary acidic protein (GFAP; 1:200, Abcam; AB4648), and mouse monoclonal anti‐BDNF (1:500, Abcam; ab203573) antibody. After washing three times in PBS, the sections were incubated with the corresponding Invitrogen Alexa Fluor secondary antibodies in PBS for 1 hr at room temperature. The sections were then washed and mounted, and coverslips were secured on the slides using a mounting medium (Vector Laboratories, Inc. Burlingame, CA; H1200). Confocal images were captured using a Nikon confocal Axiovert microscope (Carl Zeiss, Göttingen, Germany; LSM510).

### 2.9 Enzyme‐linked immunosorbent assay (ELISA) of Aβ levels

Total concentrations of Aβ40 and Aβ42 were determined using a well‐established protocol for ELISA. Aβ40 (Invitrogen; KHB3441) and Aβ42 (Invitrogen; KHB3481) ELISA kits were used in accordance with the manufacturer's instructions. The optical densities of each sample were read on a VersaMax tunable microplate reader (Molecular Devices), and the concentrations of Aβ40 and Aβ42 were determined according to the respective standard curves.

### 2.10 Immunoblotting analysis

Equal amounts of total protein samples from the hippocampus were separated via SDS/PAGE (10% Bis‐Tris gel; Invitrogen), following which they were transferred onto a PVDF membrane (Bio‐Rad Laboratories) and blocked in TBST buffer (25 mM Tris‐HCl, 160 mM sodium chloride, and 0.05% Tween‐20) containing 5% (wt/vol) bovine serum albumin (Santa Cruz Biotechnology) for 1 hr at 25°C. The membrane was then incubated with primary antibodies overnight at 4°C. This was followed by incubation with the corresponding secondary antibody for 1 hr at room temperature. Primary antibodies included mouse anti–cleaved caspase‐3 (1:400, Cell Signaling Technology), mouse anti‐Bax (1:1000, Santa Cruz Biotechnology), mouse anti‐Bcl‐2 (1:500, Santa Cruz Biotechnology), mouse anti‐p65 (1:500, Santa Cruz Biotechnology), and rabbit anti‐GAPDH (1:600, Cell signaling Technology). Immunoblot bands were visualized using an ECL Western blot kit (NeoBioscience Biotechnology, China). An Eastman Kodak Image Analyzer (Rochester, NY) was used for semiquantification of the protein signal intensity.

### 2.11 Statistical analysis

All data are presented as means ± the standard error of the mean (*SEM*). Two‐way ANOVA was used for water maze escape latency analysis. One‐way analyses of variance (ANOVA) followed by Tukey's post hoc test were used to compare data between the groups. The level of statistical significance was set at *p* < .05. All graphs were generated using GraphPad Prism 7.0 (GraphPad Software).

## 3 RESULTS

### 3.1 IH improves cognition in APP/PS1 mice

**Figure 1:**
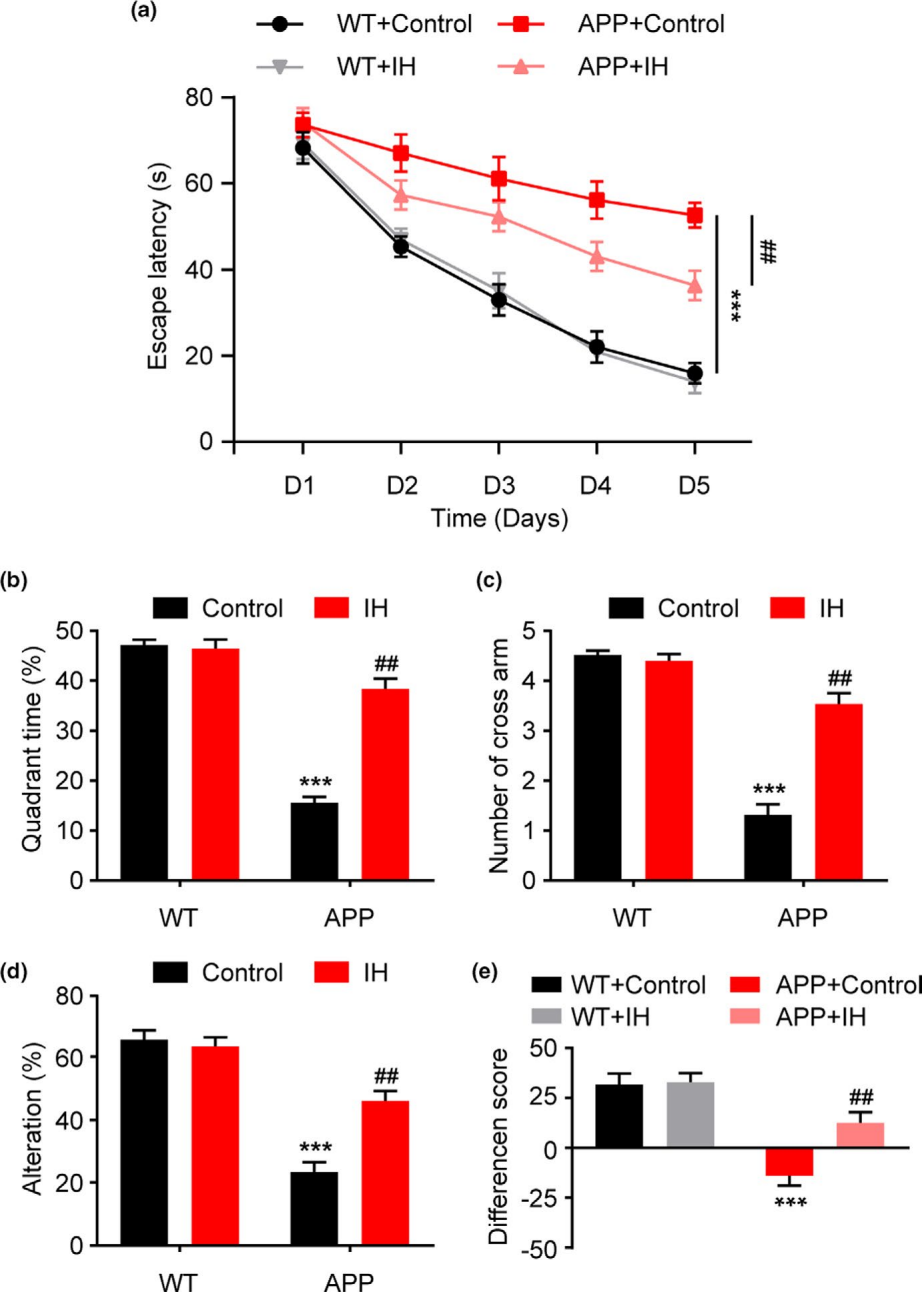
Exposure to intermittent hypoxia (IH) attenuates impairments in spatial and nonspatial memory in APP/PS1 mice. (a) Performance during the acquisition phase of the Morris water maze test. (b) The percentage of time spent in the target quadrant during the probe trial. (c) The number of times mice swam across the borders of the target quadrant. (d) Spontaneous alternation performance in the Y‐maze test. (e) Effect of IH exposure on the difference score in the NOR test. Data were compared using analyses of variance (ANOVA) followed by Bonferroni's post hoc test. ***p < .001 compared with wild‐type (WT) controls and ##*p* < .01 compared with APP/PS1 controls. *n* = 8–10 per group

At the end of the 2‐week daily IH exposure, we examined the effects of IH on hippocampus‐dependent spatial memory in wild‐type (WT) and APP/PS1 mice using the MWM test. During the acquisition test, there were no significant differences in escape latency between WT control mice and WT mice exposed to IH (Figure 1a, Groups: F3, 34 = 21.32, *p* < .0001; time: F4,136 = 28.14, *p* < .0001; groups × time: F12, 136 = 0.864, *p* > .05; two‐way ANOVA). Consistent with previous results, APP/PS1 control mice exhibited significantly longer escape latencies than WT controls, whereas IH‐exposed APP/PS1 mice exhibited significantly shorter escape latencies. Moreover, during probe trials, IH‐exposed APP/PS1 mice traveled more often and spent more time within the target quadrant than control APP/PS1 mice (Figure 1b, F3, 34 = 6.732, *p* < .001, one‐way ANOVA; Figure 1c, F3, 34 = 8.174, *p* < .0001, one‐way ANOVA). We then evaluated the effect of IH exposure using the Y‐maze test. IH‐exposed APP/PS1 mice exhibited increased spontaneous alternation compared to control APP/PS1 mice (Figure 1d, F3, 34 = 8.863, *p* < .0001, one‐way ANOVA). The NOR test was performed to evaluate short‐term nonspatial memory. After the training sessions, mice were able to distinguish the novel object, and IH exposure had little effect on the discrimination ratio in WT mice (Figure 1e, F3, 34 = 14.542, *p* < .0001, one‐way ANOVA). However, IH exposure attenuated impairments in nonspatial memory in APP/PS1 mice, as indicated by a relative increase in the preference for the novel object.

### 3.2 IH reduces anxiety‐related behaviors in APP/PS1 mice

**Figure 2:**
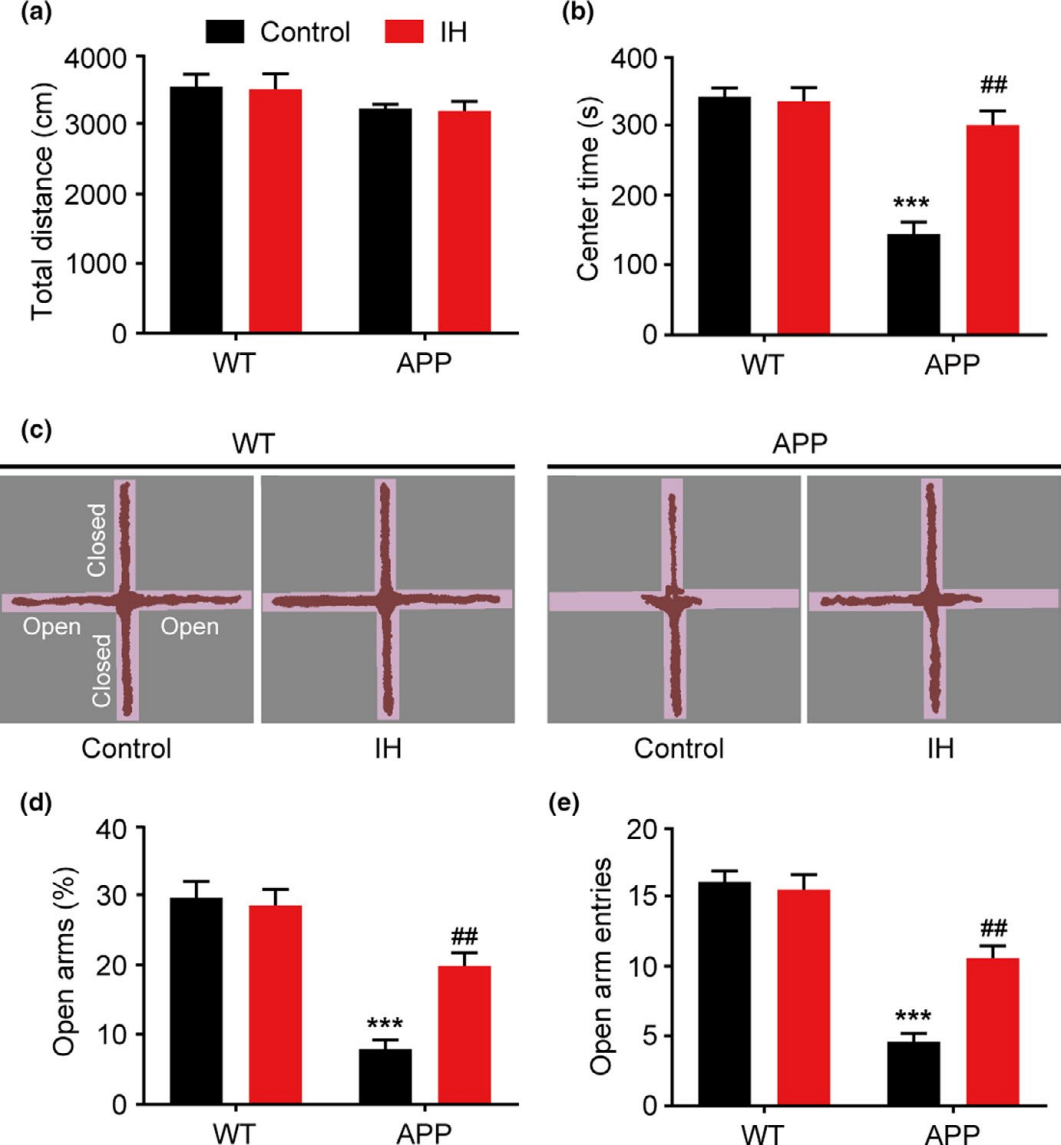
Exposure to intermittent hypoxia (IH) attenuates anxiety‐like behavior in APP/PS1 mice. (a) Distance traveled in the open‐field (OF) chamber. (b) Time spent in the central area of the OF chamber. (c) Representative movement traces during the elevated plus‐maze (EPM) test. (d) Time spent in open arms. (e) The frequency of entries into open arms. Data were compared using analyses of variance (ANOVA) followed by Bonferroni's post hoc test. ***p < .001 compared with wild‐type (WT) controls and ##*p* < .01 compared with APP/PS1 controls. *n* = 8–10 per group

We then investigated the effects of IH exposure on locomotor activity in APP/PS1 mice using the OFT. There were no significant differences in the distance traveled between the groups (Figure 2a, F3, 34 = 0.2431, *p* > .05, one‐way ANOVA). Control APP/PS1 mice were less inclined to explore the central area of the OFT chamber than WT controls (Figure 2b, F3, 34 = 6.642, *p* < .0001, one‐way ANOVA), suggestive of increases in anxiety‐related behavior. However, IH‐exposed APP/PS1 mice spent more time exploring the central area than control APP/PS1 mice. The EPM test was used to evaluate the levels of anxiety in each group. Although IH had no effect in WT mice, IH‐exposed APP/PS1 mice spent more time exploring the open arm (Figure 2c,d, F3, 34 = 9.632, *p* < .0001, one‐way ANOVA) and entered the open arm more frequently than their counterparts (Figure 2e, F3, 34 = 7.135, *p* < .0001, one‐way ANOVA), suggesting that IH attenuated anxiety‐related behaviors.

### 3.3 IH reduces Aβ deposition in the hippocampus of APP/PS1 mice

**Figure 3:**
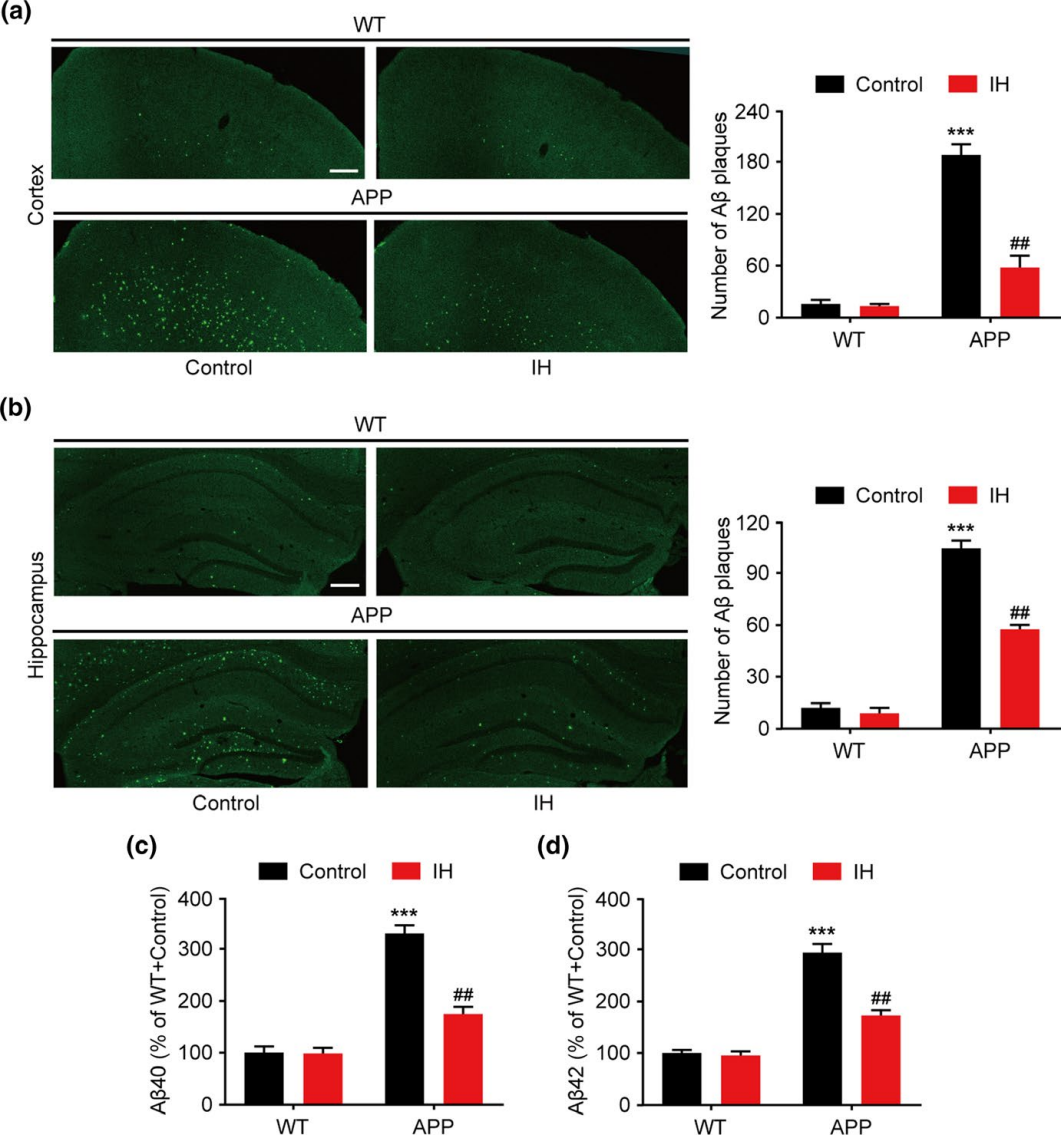
Intermittent hypoxia (IH) pretreatment reduces amyloid β (Aβ) deposition in the hippocampus of APP/PS1 mice. (a) Immunofluorescence image and the number of Aβ plaque deposits in the cortex of APP/PS1 mice (scale bar = 200 μm). (b) Immunofluorescence image and the number of Aβ plaque deposits in the hippocampus of APP/PS1 mice (scale bar = 200 μm). (c, d) Levels of total Aβ40 (c) and Aβ42 (d) were quantified using sandwich enzyme‐linked immunosorbent assays (ELISAs) and expressed as fold changes compared to wild‐type (WT) control mice. Data were compared using analyses of variance (ANOVA) followed by Bonferroni's post hoc test. ***p < .001 compared with WT controls and ##*p* < .01 compared with APP/PS1 controls. *n* = 6–8 per group

An abnormal increase in the deposition of Aβ plaques is a pathological hallmark of APP/PS1 mice. To elucidate the biochemical mechanisms underlying the effects of IH exposure in APP/PS1 mice, we assessed plaque deposits in the cortex and hippocampus using immunofluorescence. Immunopositive plaques were detected in the hippocampus of control APP/PS1 mice. IH pretreatment significantly reduced the number of plaque deposits (Figure 3a, F3, 24 = 16.647, *p* < .0001, one‐way ANOVA; Figure 3b, F3, 24 = 13.543, *p* < .0001, one‐way ANOVA;). Furthermore, sandwich ELISAs revealed that IH pretreatment significantly lowered excessive levels of Aβ40 and Aβ42 in the hippocampus of APP/PS1 mice (Figure 3c, F3, 24 = 7.523, *p* < .0001, one‐way ANOVA; Figure 3d, F3, 24 = 8.174, *p* < .0001, one‐way ANOVA).

### 3.4 IH enhances neurogenesis in the hippocampus of APP/PS1 mice

**Figure 4:**
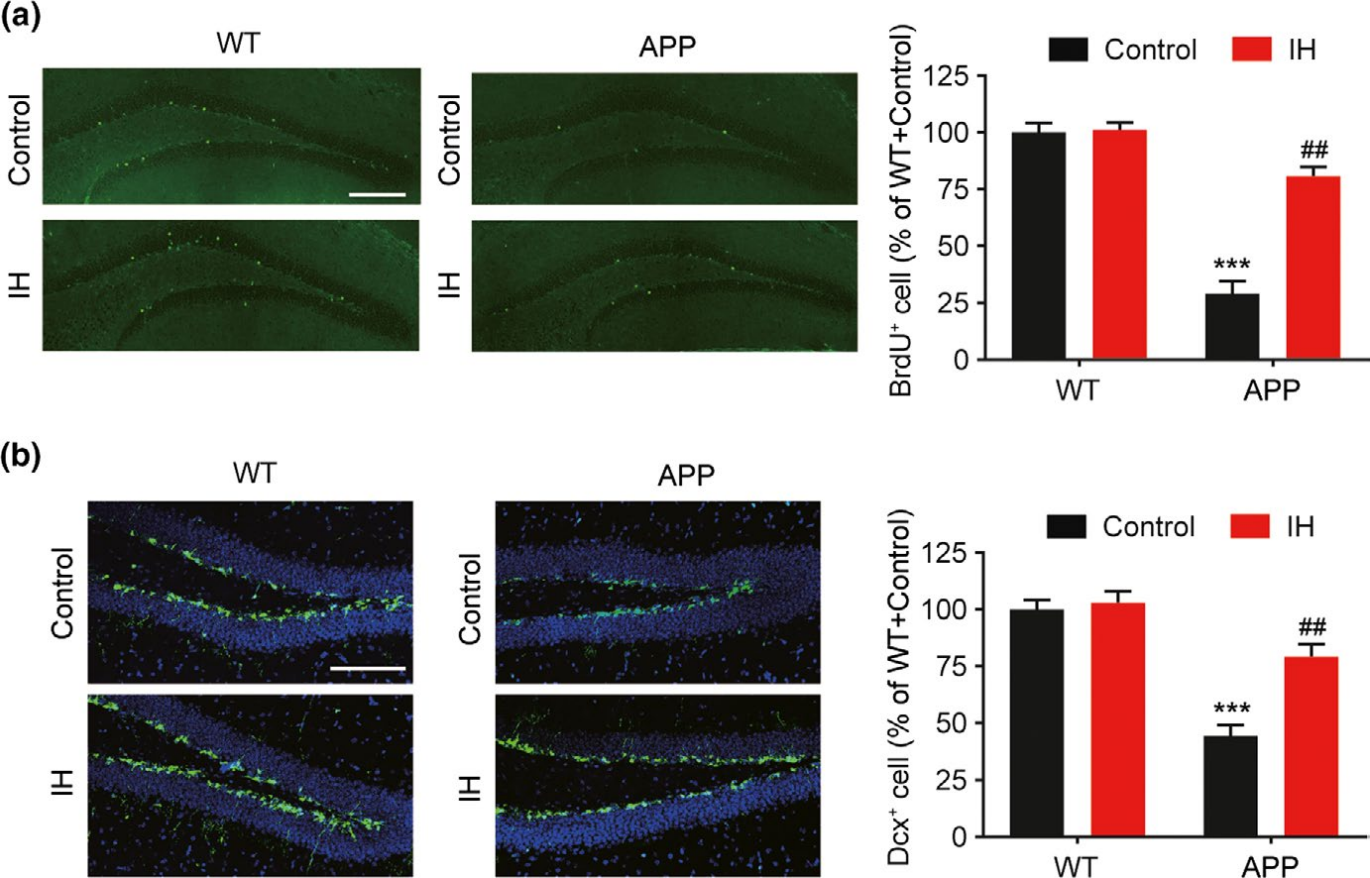
Intermittent hypoxia (IH) pretreatment promotes hippocampal neurogenesis in APP/PS1 mice. (a) Representative confocal micrographs of hippocampal sections stained for bromodeoxyuridine (BrdU) and quantification of cell numbers (scale bar = 200 μm). (b) Representative confocal micrographs of hippocampal sections stained for doublecortin (DCX) and quantification of cell numbers (scale bar = 100 μm). Data were compared using analyses of variance (ANOVA) followed by Bonferroni's post hoc test. ***p < .001 compared with wild‐type (WT) controls and ##*p* < .01 compared with APP/PS1 controls. *n* = 10 per group

Since neuroplasticity plays an important role in memory and associated learning, we evaluated neural plasticity induced by IH pretreatment. We analyzed hippocampal neurogenesis by counting mitotic (marked by BrdU) and neuronal progenitor (marked by DCX) cell numbers in the dentate gyrus (DG) of APP/PS1 mice. IH pretreatment significantly increased the number of BrdU‐ and DCX‐positive cells in the DG of IH‐treated APP/PS1 mice when compared with levels observed in control APP/PS1 mice (Figure 4a, F3, 36 = 8.926, *p* < .0001, one‐way ANOVA; Figure 4b, F3, 36 = 8.064, *p* < .0001, one‐way ANOVA). No significant alterations in neurogenesis were observed in WT mice.

### 3.5 IH enhances BDNF expression and inhibits apoptosis in the hippocampus of APP/PS1 mice

**Figure 5:**
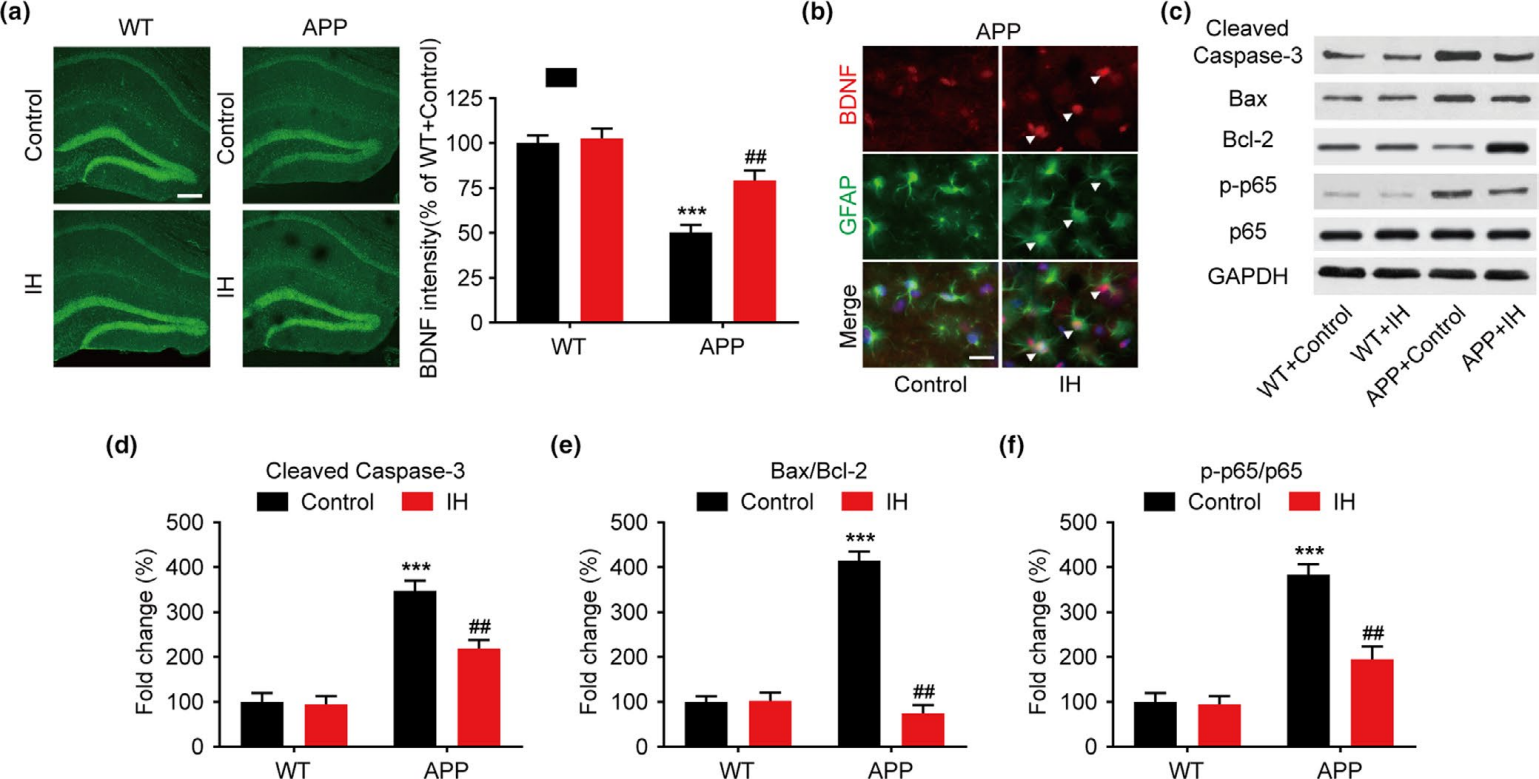
Intermittent hypoxia (IH) pretreatment promotes brain‐derived neurotrophic factor (BDNF) expression and inhibits apoptosis in the hippocampus of APP/PS1 mice. (a) Representative confocal micrographs of hippocampal sections stained for BDNF and quantified results (scale bar = 200 μm). (b) Representative confocal micrographs of hippocampal sections costained for BDNF and glial fibrillary acidic protein (GFAP). The white triangle represent colabeling (scale bar = 10 μm) (c–f) Representative Western blots showing levels of cleaved caspase‐3, Bax, Bcl‐2, p‐p65, and p65 expression, as well as internal controls, in the hippocampus of APP/PS1 mice. Data were compared using analyses of variance (ANOVA) followed by Bonferroni's post hoc test. ***p < .001 compared with wild‐type (WT) controls and ##*p* < .01 compared with APP/PS1 controls. *n* = 6–8 per group

We postulated that increases in neurogenesis were caused by increased expression of neurotrophic factors. BDNF is a member of the neurotrophin family, playing an important role in the regulation of synaptic plasticity, and previous studies have indicated that BDNF may stimulate neurogenesis in AD mice (Liu et al., 2015). Therefore, we examined BDNF levels in the hippocampus in each mouse group. Photomicrographs of immunofluorescence slices revealed that BDNF signal intensity was significantly decreased in APP/PS1 control mice compared to that in WT controls (Figure 5a, F3, 36 = 14.542, *p* < .0001, one‐way ANOVA). However, IH pretreatment significantly increased BDNF expression in APP/PS1 mice. In agreement with the well‐established fact that BDNF is mainly produced by astrocytes, the BDNF and GFAP costaining demonstrated that IH specifically increased the BDNF immunofluorescence in astrocytes (Figure 5b). Hippocampal neural apoptosis is another pathological characteristic of APP/PS1 mice. To explore the anti‐apoptotic effect of IH in APP/PS1 mice, we analyzed molecules related to apoptosis via Western blotting. IH pretreatment significantly suppressed the expression of activated caspase‐3 in the hippocampus of APP/PS1 mice compared to levels observed in the control APP/PS1 group (Figure 5c,d, F3, 24 = 13.523, *p* < .0001, one‐way ANOVA). The ratio of the autophagy‐related proteins Bax/Bcl‐2 is closely related to neural apoptosis. Thus, we examined the relative expression of Bax and Bcl‐2 in the hippocampus. IH had no effect on the ratio of Bax to Bcl‐2 in WT mice. However, IH pretreatment markedly suppressed Bax and increased Bcl‐2 expression in APP/PS1 mice (Figure 5c,e, F3, 24 = 16.764, *p* < .0001, one‐way ANOVA). Furthermore, we evaluated the activation of p65, which plays an important role in apoptotic pathways. Phosphorylation of p65 in APP/PS1 mice was significantly increased relative to that in WT controls (Figure 5c,f, F3, 24 = 14.532, *p* < .0001, one‐way ANOVA). However, IH pretreatment significantly decreased the p‐p65/p65 ratio.

## 4 DISCUSSION

As the leading cause of dementia, AD is expected to affect 96 million people by 2050 (Reitz & Mayeux, 2014). Because the causes of AD are heterogeneous and complex, it is extremely difficult to develop drugs based on a single principle for the treatment of AD in all patients. Therefore, the need for new therapeutic approaches with fewer side effects and greater efficacy remains urgent. Increasing evidence supports the protective and therapeutic effects of moderate IH on the central nervous system (Tabet de Oliveira, 2014). Moderate IH in early life can accelerate brain development and improve learning abilities (Zhang, Chen, Du, Chen, & Zhu, 2005), which is related to increased neurogenesis and synaptic plasticity. In IH preconditioning of cerebral ischemia and other diseases, preconditioning attenuates the ischemic response and effectively downregulates metabolic pathways of ischemia‐induced immune responses (Goryacheva et al., 2010; Mashina et al., 2006; Zhang, Shi, & Downey, 2015). Similar to cerebral ischemia, AD also presents with inflammation, decreased synapse formation, and cognitive impairment. This suggests that IH may induce in AD similar regulatory and protective mechanisms for neurotrophic and immunoinflammatory responses. In the present study, we examined whether IH exposure can attenuate AD symptoms in a mouse model of AD. Our results indicated that exposure to IH improves memory functions and suppresses anxiety‐related behaviors in APP/PS1 mice.

Animal models provide critical tools for examining AD interventions. APP/PS1 mice are known to develop AD‐like pathology and memory impairments (Trinchese et al., 2004). In this study, we evaluated hippocampus‐dependent spatial memory and cognitive functions, which are impaired in patients with AD, to determine the effects of IH in APP/PS1 mice. APP/PS1 models of AD reproduce deficits in both of these functions. Our findings demonstrated that 15 day of IH exposure improved the performance of APP/PS1 mice in the MWM, Y‐maze, and NOR tests.

Anxiety is a common clinical feature among patients with AD, especially in those with more severe dementia (Breitve et al., 2016). Given that up to 70% of patients with AD experience symptoms of anxiety, the need to develop effective strategies for treating anxiety in clinical practice remains urgent (Teri et al., 1999). Previous studies have reported that APP/PS1 mice also exhibit anxiety‐related behaviors (Lok et al., 2013). Considering the close relationship between hippocampal neurogenesis and affective disorders, as well as the observation that IH promotes neurogenesis in AD mice, IH may antagonize anxiety by stimulating neurogenesis. Our results indicated that IH exposure exerts anxiolytic effects in APP/PS1 mice, resulting in increased exploratory activity in the OFT and increased the open‐arm exploration in the EPM.

Pathological deposition of Aβ in the brain is a well‐known characteristic of AD (Armstrong, 1994). Aβ production is associated with impaired hippocampal synaptic plasticity, which leads to behavioral abnormalities (Gray et al., 2018). In the current study, we observed that IH exposure significantly reduced the number of Aβ plaques in the hippocampus of AD mice. The amyloid load in the brain may affect important forms of neural plasticity such as neurogenesis, which is involved in learning and memory (Lazarov & Marr, 2010). Our results indicated that recovery of cognition was correlated with increased NSC proliferation and differentiation, suggesting that IH may exert its beneficial effects by restoring neurogenesis. BDNF signaling provides a beneficial microenvironment for neurogenesis. It is reasonable to speculate that the mechanism underlying IH‐stimulated neurogenesis involves the restoration of BDNF signaling. Our results are in accordance with those of previous studies in rats showing that hippocampal BDNF expression is upregulated after 14 d of IH (Zhu et al., 2010). Hippocampal BDNF also facilitates anxiolysis (Quesseveur et al., 2013), and mice with impaired BDNF/TrkB signaling display increased levels of anxiety (Bergami et al., 2008). Taken together, these findings indicate that the anxiolytic‐like effect of IH in APP mice may occur via upregulation of the BDNF pathway.

Neuronal cell apoptosis occurs in age‐related neurodegenerative diseases (Gorman, 2008). Regulatory proteins for apoptosis are implicated in the pathological neuronal death that occurs in patients with AD (Engidawork, Gulesserian, Seidl, Cairns, & Lubec, 2001). In the present study, APP/PS1 mice exhibited enhanced expression of apoptotic proteins in the hippocampus, although IH exposure markedly suppressed apoptosis in AD mice. Caspase‐3 is a member of the caspase family, acting as the proximate mediator of apoptosis (Jänicke, Sprengart, Wati, & Porter, 1998). AD mice exhibited significant increases in cleaved caspase‐3 expression in the hippocampus, while IH exposure markedly suppressed caspase‐3 expression. Bcl‐2, which inhibits apoptosis, and Bax, which promotes apoptosis, cooperatively regulate neuronal destinies (Sun, Ma, Yang, Zhao, & Zhang, 2012). The balance of Bax/Bcl‐2 expression was altered in AD mice. However, BDNF exerted neuroprotective effects against cellular apoptosis by regulating the ratio of Bax/Bcl‐2. IH exposure significantly suppressed the expression of Bax and enhanced the expression of Bcl‐2, significantly decreasing the Bax/Bcl‐2 ratio. These findings illustrate the neuroprotective effect of IH in AD mice.

Although the impact of IH on AD patients has not been elucidated yet, preclinical IH regimens at low or medium intensity may prove to be safe and beneficial and provide a useful therapeutic strategy for effective brain protection in individuals with AD.

## 5 CONCLUSION

The present study is the first to investigate the effects of IH exposure on spatial memory, cognition, and anxiety‐related behavior in the APP/PS1 mouse model of AD. IH increased Aβ clearance and neurogenesis while decreasing neuronal apoptosis in APP/PS1 mice. These results may aid in the development of safer, more effective treatments for AD, and other disorders associated with memory impairments and anxiety‐like symptoms.

## CONFLICT OF INTEREST

The authors declare that they have no conflict of interest.

## Data Availability

All data used during the study are available from the corresponding author by request.
